# A robust MR-based attenuation map generation in short-TE MR images of the head employing hybrid spatial fuzzy C-means clustering and intensity inhomogeneity correction

**DOI:** 10.1186/2197-7364-1-S1-A49

**Published:** 2014-07-29

**Authors:** Mohammad Hadi Aarabi, Anahita Fathi Kazerooni, Parisa Khateri, Mohammad Reza Ay, Hamidreza Saligheh Rad

**Affiliations:** Quantitative MR Imaging and Spectroscopy Group, Research Center for Cellular and Molecular Imaging, Tehran University of Medical Sciences, Tehran, Iran; Department of Medical Physics and Biomedical Engineering, School of Medicine, Tehran University of Medical Sciences, Tehran, Iran; Medical Imaging Systems Group, Research Center for Cellular and Molecular Imaging, Tehran University of Medical Sciences, Tehran, Iran

Deriving an accurate attenuation correction map (μ-map) from magnetic resonance (MR) volumes has become an important problem in hybrid PET/MR imaging. Recently, short echo-time (STE) MR imaging technique incorporating fuzzy C-means (FCM) tissue classification and 2-point Dixon image acquisition has been introduced as a feasible approach for segmentation of the bone from air and soft tissue. However, this method imposes additional imaging and the performance of the standard FCM algorithm, suffering from the lack of spatial information, becomes impaired in the presence of inherent noise and intensity inhomogeneity. Here, we exploit a spatial fuzzy C-means (SFCM) segmentation algorithm in combination with a robust intensity inhomogeneity correction method on single STE-MR images, to differentiate various tissue classes.

MR images of five subjects were acquired on a clinical 1.5T MRI System, MAGNETOM Avanto, using a FLASH 3D pulse sequence with *TE*=1.1*ms*, *TR*=12*ms*, *flip angle*=18°, *voxel size*=1.2×1.2×2*mm*^3^.

The proposed segmentation approach consists of four main steps: (1) Intensity-inhomogeneity correction, to separate air and bone in the regions with high inhomogeneity, like the nasal areas; (2) applying SFCM to segment the images into four clusters including air, a part of soft tissue and bone, and two other soft tissue classes. Upon this step, the air cluster would be accurately separated; (3) employing shape factor analysis to remove the eyes with close signal intensity to that of bone; and (4) μ-map generation by downsampling and assigning attenuation coefficients to the corresponding segmented tissues.

Quantitative evaluation indicated sensitivity of over 95% for air and soft tissue and about 81% in the bony region, and specificity of over 95% for all tissue classes. The proposed STE-MR imaging in combination with the segmentation technique could be potentially exploited as an efficient approach to generate MR-based attenuation correction maps in clinical PET/MR applications, where bone and air coexist.

